# Coexisting Pyogenic and Tuberculous Liver Abscess in Uncontrolled Diabetes: A Diagnostic Challenge

**DOI:** 10.1002/ccr3.73149

**Published:** 2026-07-14

**Authors:** Hamna Mehfooz, Azba Israr, Syed Hassan Tanvir Ramzi, Akram Malik, Syed Muhammad Salman Hassan, Sarim Hassan Shahab, Fazal Habib

**Affiliations:** ^1^ Department of Medicine Mukhtar A. Sheikh Hospital Multan Pakistan; ^2^ Bakhtawar Amin Medical and Dental College Multan Pakistan; ^3^ The Cancer Clinic Multan Pakistan; ^4^ Nishtar Medical University Multan Pakistan; ^5^ Sher‐e‐Bangla Medical College Barisal Bangladesh

**Keywords:** diabetes mellitus, GeneXpert, hepatic tuberculosis, liver abscess, *Mycobacterium tuberculosis*

## Abstract

Hepatic tuberculosis can mimic pyogenic liver abscess, particularly in patients with uncontrolled diabetes. Persistent or atypical hepatic lesions unresponsive to antibiotics should prompt tissue diagnosis using histopathology and molecular assays like GeneXpert to ensure timely diagnosis and appropriate management.

## Key Learning Point

Persistent or atypical liver abscesses unresponsive to standard antibiotics should prompt early tissue diagnosis using histopathology and molecular testing to exclude hepatic tuberculosis, even in the absence of pulmonary disease.

## 1 Introduction

Tuberculosis (TB) remains a major global health problem, particularly in endemic regions despite advances in diagnosis and treatment [1]. Extrapulmonary tuberculosis accounts for nearly 15%–20% of TB cases in immunocompetent individuals and up to 50% in immunocompromised patients [2]. Hepatic involvement is uncommon and may occur as diffuse hepatic infiltration, miliary disease, or localized tuberculous liver abscess, the latter being particularly rare [3, 4]. Hepatic TB may result from hematogenous dissemination, direct extension from adjacent lymph nodes, or reactivation of latent hepatic foci [4, 5, 6].

Clinical presentation is usually nonspecific and may include fever, abdominal pain, malaise, and weight loss [7]. Radiological findings often mimic pyogenic or amoebic liver abscesses as well as hepatic malignancies, making diagnosis difficult [8, 9]. Common investigations include ultrasonography, computed tomography (CT), microbiological cultures, histopathology, and molecular assays such as GeneXpert, which are important in establishing the diagnosis [4, 8, 10].

Certain conditions, such as diabetes mellitus, increase susceptibility to both tuberculosis and secondary bacterial infections because of impaired cellular immunity and altered immune response [11, 12, 13]. We report a rare case of coexisting pyogenic and tuberculous liver abscess in a patient with poorly controlled diabetes mellitus, highlighting the diagnostic challenges and importance of tissue diagnosis in persistent hepatic lesions.

## 2 Case History, Differential Diagnosis, Investigations and Treatment

A 47‐year‐old male poultry farm owner, father of four, and known case of poorly controlled diabetes mellitus on biphasic human insulin therapy presented with right hypochondrial pain for one and a half months, associated with approximately 5‐kg weight loss. He also reported a preceding episode of fever and cough that resolved before presentation. His past medical history was significant for chronic hepatitis C, for which he had completed antiviral therapy three months earlier with sustained virological response. He also had a history of laparoscopic cholecystectomy performed six years earlier for cholelithiasis.

On examination, the patient appeared mildly cachectic with a body mass index suggestive of mild malnutrition. He was afebrile and hemodynamically stable. Mild tenderness was noted in the right upper quadrant without organomegaly or palpable masses. Laboratory investigations revealed poor glycemic control with HbA1c of 11.6%.

**FIGURE 1:**
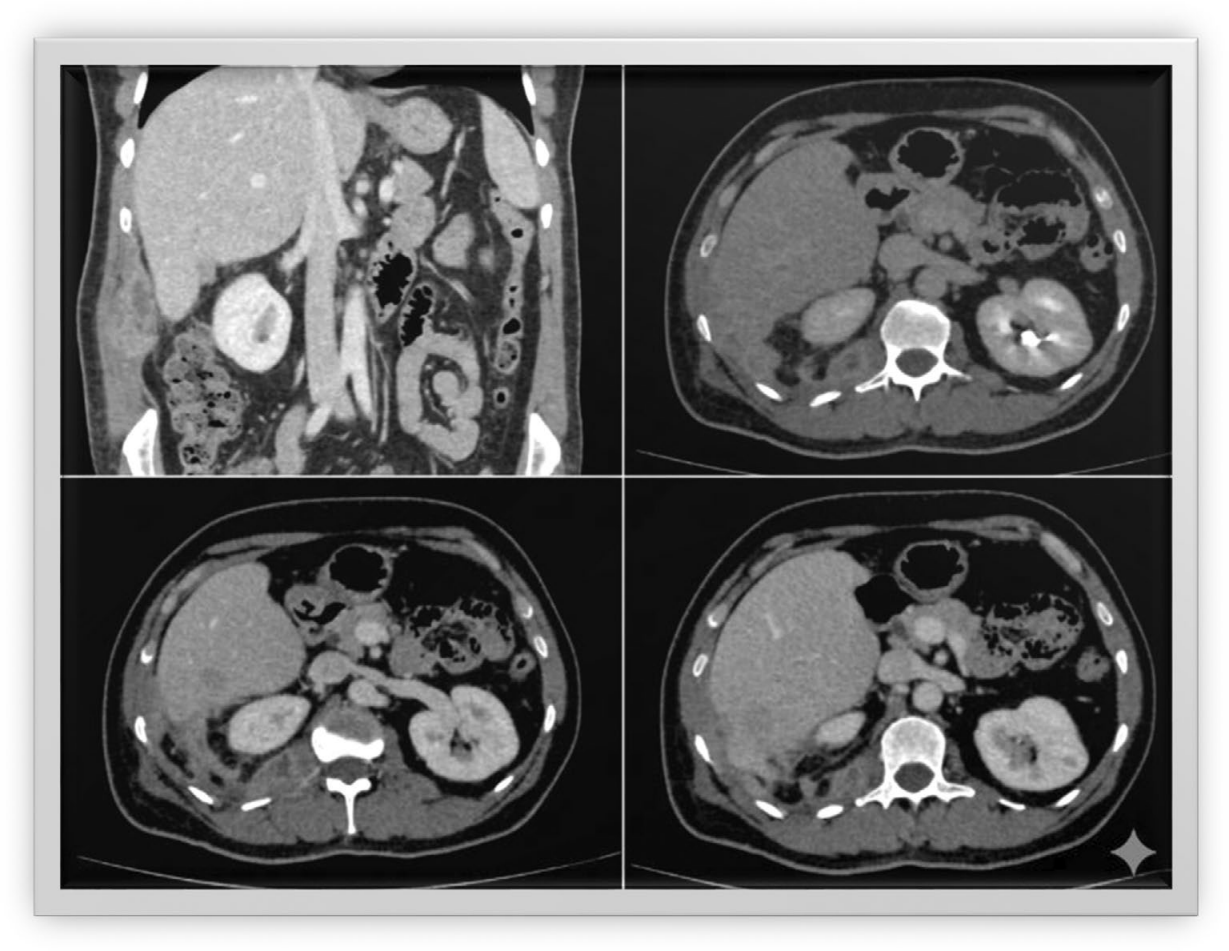
Contrast‐enhanced computed tomography (CT) of the abdomen at presentation demonstrating two hypodense lesions within the right hepatic lobe, consistent with developing liver abscesses. Coronal (upper left) and axial (upper right, lower left, and lower right) images show irregular low‐attenuation lesions with surrounding inflammatory changes. Serial follow‐up imaging demonstrated only partial interval regression despite culture‐directed antibiotic therapy, prompting further diagnostic evaluation for an atypical infection.

Initial contrast‐enhanced CT abdomen (CT‐1) demonstrated two hypodense hepatic lesions suggestive of developing liver abscesses. Serial CT and ultrasound examinations performed over the following weeks showed partial but incomplete interval regression of the lesions, following antibiotic therapy, with persistent right lobe hepatic lesions and surrounding inflammatory changes, raising suspicion for atypical or mixed infective pathology. (Figure 1).

Despite antimicrobial therapy, the patient continued to complain of persistent right upper abdominal pain, poor appetite, easy fatigability, and ongoing weight loss, although the initial febrile illness had subsided. In view of the incomplete symptomatic improvement and persistent hepatic lesions, serial CT and ultrasound examinations were performed during follow‐up to assess treatment response, evaluate lesion progression, identify possible complications, and guide further management. Repeated imaging consistently demonstrated residual right hepatic lobe lesions with surrounding inflammatory changes and only partial interval resolution despite culture‐directed antibiotic therapy. These persistent radiological abnormalities, together with ongoing constitutional symptoms, raised suspicion for an atypical or mixed infective etiology, including granulomatous disease, and therefore prompted repeat aspiration and ultrasound‐guided tissue biopsy for definitive diagnosis.

Differential diagnoses included a pyogenic liver abscess, an amoebic abscess, hepatic tuberculosis, and less likely, a primary or metastatic hepatic malignancy.

Ultrasound‐guided aspiration yielded reddish pus, which on culture demonstrated a moderate growth of *Escherichia coli* sensitive to amoxicillin–clavulanate, piperacillin–tazobactam, carbapenems, and aminoglycosides. Due to incomplete radiological resolution, repeat aspiration and ultrasound‐guided trucut biopsy of the hepatic lesion were performed.

**FIGURE 2:**
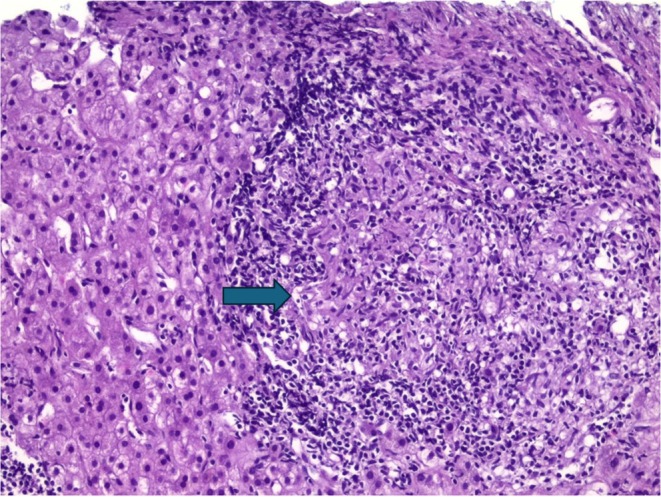
Liver parenchyma showing a granuloma with a collection of epithelioid histiocytes surrounded by a cuff of lymphocytes (H&E x 40).

**FIGURE 3:**
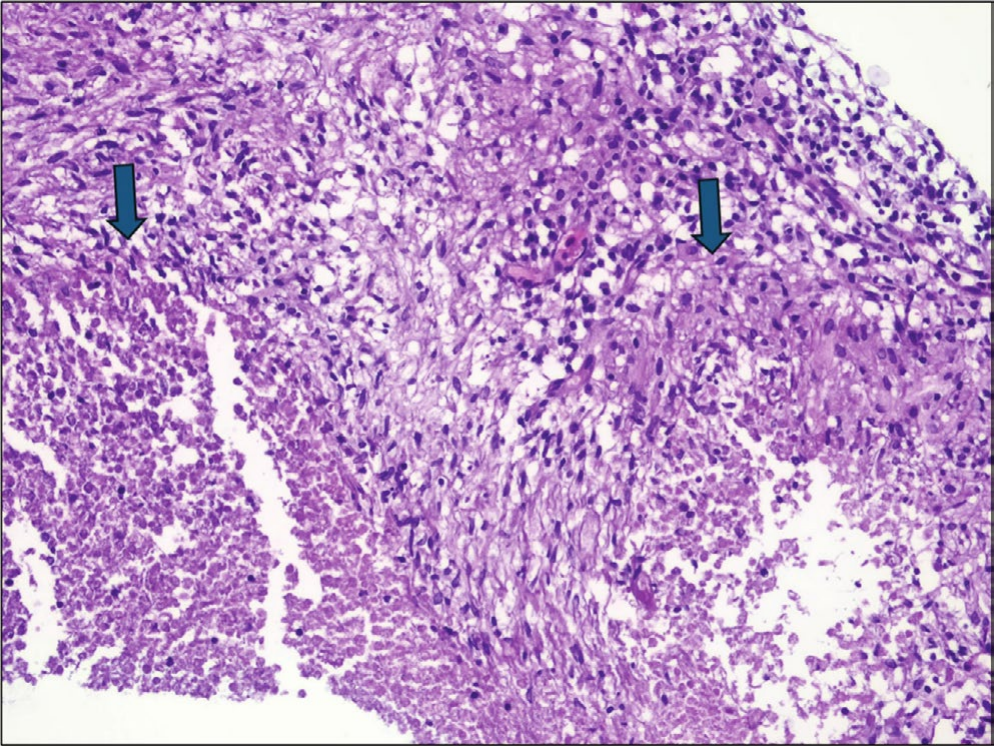
Liver biopsy showing an area with caseation necrosis surrounded by palisading granulomatous inflammation (H&E x 40).

The GeneXpert assay detected *Mycobacterium tuberculosis* complex at a trace level, while rifampicin resistance was indeterminate. Acid‐fast bacilli smear was negative. Histopathological examination revealed granulomatous inflammation composed of epithelioid histiocytes with surrounding lymphocytic cuffing along with areas of caseation necrosis, consistent with tuberculous granulomatous hepatitis (Figures 2 and 3).

During treatment, the patient developed antituberculous therapy‐induced hepatitis approximately three weeks after initiation of therapy. Liver function tests showed an elevation of serum alanine aminotransferase (ALT) to 286 U/L, aspartate aminotransferase (AST) to 241 U/L, and total bilirubin to 2.1 mg/dL, consistent with drug‐induced hepatotoxicity. Consequently, isoniazid, rifampicin, and pyrazinamide were withheld. A modified regimen consisting of levofloxacin, ethambutol, and aminoglycoside‐based injectable therapy was initiated. Following improvement in liver function tests, low‐dose isoniazid was cautiously reintroduced with close biochemical monitoring. The patient showed significant symptomatic and radiological improvement during follow‐up.

## 3 Results and Conclusion

A prolonged antituberculous treatment course of 9 months was planned because of hepatic tuberculosis with poorly controlled diabetes mellitus and delayed radiological resolution. Treatment response was assessed through serial clinical improvement, normalization of liver function tests, and follow‐up ultrasound and CT imaging, which demonstrated a progressive reduction in the hepatic lesions and surrounding inflammatory changes. (Tables 1 and 2).

**TABLE 1:** Timeline diagram demonstrating the clinical course, serial imaging findings, microbiological investigations, biopsy results, initiation of antituberculous therapy, development of hepatotoxicity, and subsequent clinical improvement during follow‐up.

Timeline of clinical course and investigations							
Symptom onset	CT‐1 abdomen	Serial CT/ultrasound	Aspiration and culture	Biopsy and GeneXpert	Antituberculous therapy started	ATT‐induced hepatitis	Follow‐up
Right hypochondrial pain, fever, cough and~5 kg weight loss Duration: ~1.5 months	Two hypodense hepatic lesions suggestive of developing abscesses	Partial but incomplete interval regression of lesions after antibiotic therapy	Ultrasound‐guided aspiration yielded reddish pus Culture: Moderate growth of *Escherichia coli*	Trucut biopsy performed Histopathology: Granulomatous inflammation with caseation necrosis GeneXpert: MTB complex detected (Trace) AFB smear: Negative	Started on first‐line ATT: Isoniazid Rifampicin Pyrazinamide Ethambutol	Development of hepatitis during treatment Withheld Isoniazid, Rifampicin and Pyrazinamide Switched to Levofloxacin + Ethambutol + Aminoglycoside (injectable)	Improvement in liver function tests Low‐dose Isoniazid reintroduced cautiously Significant symptomatic and radiological improvement
0 Weeks	At presentation	2–4 Weeks	4–6 Weeks	6–8 Weeks	8–9 Weeks	10–12 weeks	Follow‐up (3–6 months)
**Summary of events**							
Clinical presentation	CT‐1: hepatic lesions detected	Partial regression on imaging	Aspiration: *E. coli* on culture	Biopsy and GeneXpert: TB confirmed	ATT initiated	ATT‐induced hepatitis and regimen modification	Clinical and radiological improvement

Abbreviations: AFB: acid‐fast bacilli; ATT: antituberculous therapy; MTB: mycobacterium tuberculosis.

**TABLE 2:** Laboratory, microbiological, and histopathological findings during diagnosis and follow‐up of a patient with coexisting pyogenic and tuberculous liver abscess.

Parameter	At admission	During follow‐up/ATTIH	Clinical significance
**HbA1c**	**11.6%**	—	Poor glycemic control; increased susceptibility to TB and secondary bacterial infection
**Serum creatinine**	**0.948 mg/dL**	Stable	Preserved renal function
**eGFR**	**> 60 mL/min/1.73 m** ^**2**^	Stable	Normal renal function
**ALT (alanine aminotransferase)**	Initially near baseline	**286 U/L**	Elevated during antituberculous therapy; suggestive of drug‐induced hepatotoxicity
**AST (aspartate aminotransferase)**	Initially near baseline	**241 U/L**	Elevated during antituberculous therapy
**Total bilirubin**	Initially normal	**2.1 mg/dL**	Hyperbilirubinemia associated with hepatotoxicity
**Complete blood count**	Mild inflammatory changes	Improved on follow‐up	Supportive of resolving infection
**Liver function tests**	Baseline liver dysfunction absent	Improved after withdrawal of hepatotoxic ATT	Biochemical recovery following regimen modification
**Urine routine examination**	No major abnormality detected	—	Supportive baseline evaluation
**Pus culture from liver abscess**	Growth of *Escherichia coli* , sensitive to amoxicillin–clavulanate, piperacillin–tazobactam, carbapenems, and aminoglycosides; trace‐positive for *Mycobacterium tuberculosis* complex, rifampicin resistance indeterminate	—	Confirmed superimposed pyogenic infection
**GeneXpert MTB/RIF**	*Mycobacterium tuberculosis* complex detected; rifampicin resistance indeterminate	—	Confirmed hepatic tuberculosis
**AFB smear**	Negative	—	Consistent with paucibacillary extrapulmonary TB
**Histopathology**	Granulomatous inflammation with caseation necrosis	—	Diagnostic of tuberculous granulomatous hepatitis

Hepatic tuberculosis (TB) presenting as a liver abscess is an uncommon but important diagnostic consideration, particularly in endemic regions and in patients with immunocompromising conditions such as diabetes mellitus and malnutrition. This case highlights the critical role of histopathology and molecular assays like GeneXpert in identifying *Mycobacterium tuberculosis* in atypical hepatic lesions, especially when imaging and culture findings are inconclusive. Early recognition and timely initiation of antituberculous therapy can prevent complications and avoid unnecessary invasive procedures. Clinicians should maintain a high index of suspicion for hepatic TB in patients with non‐resolving liver abscesses and remain vigilant for potential antituberculous drug‐induced hepatotoxicity during treatment.

## 4 Discussion

Hepatic tuberculosis (TB) is a rare extrapulmonary manifestation of *Mycobacterium tuberculosis* , accounting for less than 1% of all TB cases and nearly 9% of abdominal TB presentations [14, 15]. Hepatic involvement may occur in miliary, diffuse, or localized abscess form, the latter being particularly uncommon in immunocompetent individuals [16, 17]. Because its clinical and radiologic features often overlap with pyogenic or amoebic liver abscesses and hepatic malignancies, diagnosis remains challenging [18].

In the present case, uncontrolled diabetes mellitus (HbA1c 11.6%), cachexia, and mild malnutrition were likely important predisposing factors for TB. Malnutrition impairs cell‐mediated immunity and granuloma formation, thereby increasing susceptibility to active TB [19, 20]. Diabetes is similarly associated with altered cytokine responses, impaired macrophage function, and defective cellular immunity, increasing vulnerability to both TB and secondary bacterial infections [11, 12, 13, 21]. The coexistence of *Mycobacterium tuberculosis* and *Escherichia coli* in this patient therefore reflects the increased risk of polymicrobial infection in diabetic and nutritionally compromised individuals.

Serial CT and ultrasound imaging demonstrated persistent right hepatic lobe lesions with incomplete regression despite antibiotic therapy, raising suspicion for an atypical infection. Histopathology ultimately demonstrated granulomatous inflammation with caseation necrosis, while GeneXpert detected *Mycobacterium tuberculosis* complex at a trace level, confirming hepatic TB. The low PCR bacillary load despite a tuberculous abscess may be explained by the paucibacillary nature of extrapulmonary TB, where dense granulomatous inflammation limits the organism burden [10]. Extrapulmonary specimens generally contain fewer bacilli, which reduces the molecular detection yield even in confirmed disease [10]. Prior empirical antimicrobial exposure may also have contributed to reduced mycobacterial load before tissue diagnosis. Concurrent growth of *E. coli* from aspirated pus confirmed superimposed pyogenic infection, a rare but recognized phenomenon [22, 23]. Several previous case reports and series have highlighted the diagnostic difficulty of hepatic tuberculosis because of its nonspecific clinical and radiological presentation. A review of 29 cases demonstrated that fever, abdominal pain, and hypodense hepatic lesions were common but nonspecific findings, frequently delaying diagnosis [3]. Another reported case described a primary tubercular liver abscess in an immunocompetent adult that mimicked a pyogenic liver abscess radiologically and required histopathological confirmation [18]. A mixed pyogenic and tuberculous liver abscess has also been reported, where persistent symptoms despite antibiotic therapy prompted further evaluation for tuberculosis [23].

The diagnosis of hepatic TB remains difficult because radiologic findings may mimic pyogenic abscesses, metastatic disease, or a primary hepatic malignancy [8, 14, 18]. Therefore, tissue diagnosis with histopathology and molecular assays such as GeneXpert is crucial, especially in lesions that fail to respond to conventional antimicrobial therapy. In this patient, the combination of characteristic histopathological findings and trace‐positive GeneXpert established the diagnosis despite negative acid‐fast bacilli smear.

Treatment generally follows standard antituberculous therapy (ATT) regimens [24]. However, TB‐diabetes comorbidity is associated with a delayed treatment response, an increased relapse risk, and higher mortality [20]. Patients with diabetes also demonstrate a higher risk of drug‐resistant tuberculosis and poorer treatment outcomes because of impaired host immunity, higher bacillary burden, and altered pharmacokinetics of antituberculous drugs [25]. Consequently, prolonged treatment duration up to 9 months may be considered in selected extrapulmonary TB patients with poorly controlled diabetes. Rifampicin is a potent hepatic enzyme inducer and may reduce the efficacy of oral hypoglycemic agents, necessitating close glucose monitoring and adjustment of diabetic therapy during ATT [26]. Emerging evidence suggests that metformin may improve TB outcomes through host‐directed immunomodulatory effects, including enhanced autophagy and macrophage‐mediated mycobacterial clearance [27, 28].

A major complication in this patient was antituberculous therapy‐induced hepatitis (ATTIH), which developed three weeks after treatment initiation. ATT‐related hepatotoxicity occurs in approximately 2%–28% of patients [29, 30, 31]. Underlying hepatic involvement, malnutrition, diabetes, prior liver disease, and polypharmacy may further increase susceptibility to hepatotoxicity [29, 30, 32]. In this patient, prior hepatitis C infection, hepatic involvement, cachexia, and uncontrolled diabetes likely increased susceptibility to hepatotoxicity. Temporary withdrawal of hepatotoxic agents and initiation of a modified regimen with levofloxacin, ethambutol, and aminoglycoside‐based therapy resulted in biochemical improvement, following which first‐line agents were cautiously reintroduced.

During follow‐up, the patient showed clinical and biochemical improvement with the recovery of liver function tests. A prolonged ATT course of 9 months was planned because of hepatic TB with poorly controlled diabetes and the initially complicated clinical course.

This case highlights the diagnostic complexity of persistent hepatic abscesses in TB‐endemic regions where diabetes and malnutrition are prevalent. Hepatic TB should be considered in non‐resolving liver abscesses despite appropriate antibiotics, even in the absence of pulmonary disease. Early tissue diagnosis using histopathology and molecular testing is essential for timely treatment, while careful monitoring for ATT‐induced hepatotoxicity remains critical in patients with underlying metabolic or hepatic risk factors.

## Author Contributions

**Syed Hassan Tanvir Ramzi:** writing – original draft, writing – review and editing. **Fazal Habib:** writing – review and editing. **Hamna Mehfooz:** conceptualization, project administration, writing – original draft, writing – review and editing, supervision. **Azba Israr:** writing – original draft, writing – review and editing. **Sarim Hassan Shahab:** writing – original draft, writing – review and editing. **Akram Malik:** writing – original draft, writing – review and editing. **Syed Muhammad Salman Hassan:** writing – review and editing, writing – original draft.

## Funding

The authors have nothing to report.

## Ethics Statement

The authors have nothing to report.

## Consent

Written informed consent was obtained from the patient for publication of this case report and accompanying images.

## Conflicts of Interest

The authors declare no conflicts of interest.

## Data Availability

Data sharing not applicable to this article as no datasets were generated or analysed during the current study.
